# The Knowledge, Attitude, and Practice of Nurses Regarding Trauma-Informed Care for Traumatic Injured Patients: A Multicenter Cross-Sectional Study

**DOI:** 10.1155/jonm/2449177

**Published:** 2025-11-25

**Authors:** Lingxiao He, Yunting Luo, Jun Chen, Renrong Gong, Dengbin Liao

**Affiliations:** ^1^Trauma Center of West China Hospital /West China School of Nursing, Sichuan University, Chengdu 610041, China; ^2^Center of Infectious Diseases, West China Hospital/West China School of Nursing, Sichuan University, Chengdu 610041, China; ^3^Department of Nursing, Chengdu Xinjin District Hospital of Traditional Chinese Medicine, Chengdu 611430, China; ^4^Department of Surgery, West China Hospital/ West China School of Nursing, Sichuan University, Chengdu 610041, China

**Keywords:** cross-sectional study, knowledge, attitude and practice, trauma center, trauma-informed care, traumatic injuries

## Abstract

**Background:**

Traumatic injured patients frequently present with both acute physical injuries and the exacerbation of preexisting trauma events, substantially compromising their mental health and quality of life. Trauma-informed care is a supportive intervention approach that requires understanding of trauma experiences encountered by patients. Nurses must follow the principles of trauma-informed care during interactions with patients to improve clinical outcomes. However, the current status of knowledge, attitude, and practice regarding trauma-informed care among nurses in trauma centers has not been fully investigated.

**Objective:**

To investigate the current status of knowledge, attitude, and practice regarding trauma-informed care among nurses caring for traumatic injured patients in trauma center–certified hospitals and to identify potential influencing factors.

**Design:**

Cross-sectional survey.

**Settings and Participants:**

Nurses from 11 trauma center–certified hospitals in Sichuan Province, China.

**Method:**

The assessment was conducted using standardized instruments, including a demographic survey, a trauma-informed care knowledge, attitude, and practice (TIC-KAP) questionnaire, Interpersonal Reactivity Index-Chinese version (IRI-C), and Organizational Climate Scale for Nursing (OCSN). Univariate analysis and subsequent multiple linear regression analysis were used to explore associations between TIC-KAP and other factors.

**Results:**

A total of 304 individuals participated in the survey, with 267 valid responses. Multiple linear regression analysis identified several significant predictors of TIC-KAP scores: work in emergency department, prior knowledge of trauma-informed care, psychological trauma nursing training experience, dedicated psychological nursing positions, IRI-C scores, and OCSN scores.

**Conclusion:**

Although the participating nurses demonstrated favorable scores in trauma-informed care attitude and practice, significant gaps persist in knowledge domains, with emergency nurses exhibiting particularly notable deficiencies. The implementation of trauma-informed care could be promoted through several strategic interventions: engagement in trauma-informed care or psychological nursing training programs, establishment of dedicated psychological nursing positions, and improvement of both empathy level among nurses and organizational climate.

## 1. Introduction

Traumatic events may encompass physical injuries, violent conflicts, sexual assault, emotional abuse, and systemic or societal trauma [1]. According to the Substance Abuse and Mental Health Services Administration (SAMHSA), trauma occurs when an individual experiences one or multiple events, or a specific environment, that are perceived as life-threatening, resulting in adverse effects on the individual's functioning and mental, physical, social, emotional, or spiritual well-being [2]. Trauma can lead to persistent psychological stress, causing a range of consequences including mood disorders, posttraumatic stress disorder (PTSD), substance abuse, and other conditions that significantly impact an individual's physical and mental health as well as quality of life [3, 4], representing a global health concern.

According to a survey conducted by the World Health Organization, approximately 70.4% of people worldwide have experienced some form of traumatic event during their lifetime [5]. Among those who have experienced trauma, the prevalence of PTSD is about 5.6%, while the global population's PTSD rate is approximately 3.9% [6]. Physiological trauma, such as traumatic injuries, is a significant component of these events. Patients with such injuries often experience acute trauma while also having a history of previous traumatic events, resulting in higher psychological stress levels compared to the general population [7]. A cohort study of trauma patients in China revealed that, on average, trauma patients had experienced two previous traumatic events, with the incidence of PTSD following traumatic injury estimated at around 7.63% [8]. In a major trauma cohort in New Zealand, 34% of patients reported experiencing anxiety or depression within 6 months posttrauma [9], and the incidence of PTSD varied from 8.0% to 45.0% [10]. In the United States, over 20% of trauma survivors continue to exhibit PTSD-related symptoms 1 year after the trauma [11].

The management of traumatic injuries typically demands rapid response and life-saving interventions [12]. During initial treatment, damage control resuscitation (DCR) is prioritized, followed by definitive care once the patient is stabilized. While physical injuries receive immediate attention, psychological trauma is frequently overlooked—despite extensive evidence demonstrating its profound and enduring impact. Although the American College of Surgeons' Committee on Trauma (ACSCOT) has recommended routine screening for psychological sequelae in trauma patients [13], a nationwide survey in the United States revealed that only 27.9% of trauma centers routinely screened for and intervened in PTSD, and 38.3% of trauma centers did so for depression [14]. In China, the absence of routine PTSD screening for trauma patients and the lack of quality indicators for psychological screening [15] remain significant gaps in current clinical practice. Research shows that trauma recidivism rate was 17.1% when trauma history was sourced solely from records, but 36.6% when reported directly by patients [16]. The underreporting of trauma history by patients during clinical encounters underscores the need for universal trauma-informed care (TIC) implementation [17].

The concept of TIC was first introduced by Harris and Fallot [18], who emphasized the importance of considering patients' life contexts and histories of psychological trauma within healthcare settings. SAMHSA has further defined TIC around six key principles: (1) safety; (2) trustworthiness and transparency; (3) peer support; (4) collaboration and mutuality; (5) empowerment, voice, and choice; and (6) cultural, historical, and gender issues [2]. By implementing TIC measures, healthcare providers can deliver targeted interventions based on a thorough understanding of patients' traumatic experiences. This approach aims to reduce psychological trauma symptoms, prevent retraumatization, and improve overall health outcomes [19–21].

As frontline healthcare providers who have the most frequent contact with patients, nurses deliver direct TIC, which encompasses trauma screening, trauma-informed nursing interventions, and fostering resilience in patients [22, 23]. Consequently, nurses' adequate knowledge of and positive attitudes toward TIC are critical for promoting trauma-informed clinical practice, thereby enabling the delivery of compassionate, patient-centered care and improving patient outcomes. Nonetheless, there is still a lack of studies focusing on trauma-informed care knowledge, attitude, and practice (TIC-KAP), particularly in acute care settings. Previous research reports conflicting associations between nurses' demographic factors (age, gender, and education level) and TIC-KAP [22, 24]. However, evidence consistently demonstrates that TIC training positively influences TIC-KAP [25, 26]. Significantly, time constraints and resource scarcity constitute major barriers to TIC implementation [27, 28]. Furthermore, key organizational characteristics including resource allocation, leadership support, institutional culture, and work climate have been empirically linked to variation in TIC practices [29, 30]. Finally, establishing dedicated psychological nurse positions at the organizational level to provide psychological care and counseling services for the general population may be an effective strategy [31]. Nurses in these roles typically require a certified psychotherapist qualification, ensuring they possess standardized training in advanced counseling and psychotherapeutic techniques. However, whether the creation of such positions can enhance nurses' TIC-KAP remains an area requiring further investigation.

This study aims to understand the knowledge, attitude, and practice of nurses in Chinese trauma center–certified hospitals regarding TIC when caring for patients with traumatic injuries and analyze the influencing factors. These findings will provide references for improving nurses' knowledge levels and promoting good TIC practice in the future.

## 2. Methods

### 2.1. Study Design

This study employs a descriptive, cross-sectional design and adheres to the Strengthening the Reporting of Observational Studies in Epidemiology (STROBE) guidelines [32] for reporting observational research (see Appendix 1).

### 2.2. Study Participants

This study employed a convenience sampling method. From September to November 2024, based on hospital information obtained from the Chinese Trauma Rescue Alliance, nurses were recruited from 11 trauma center–certified hospitals in Sichuan Province. A representative sampling strategy was employed, selecting four hospitals from Northern Sichuan, four from Southern Sichuan, and three from Central Sichuan to ensure geographical coverage. We selected hospitals with trauma center certification for investigation due to the unique hybrid nature of trauma centers in China: some operate as distinct clinical units, while others function as virtual platforms coordinating multidisciplinary resources. Regardless of modality, certification ensures standardized trauma care for patients. Thus, by investigating nurses in trauma center–certified hospitals, this study captures TIC-KAP within settings adhering to standardized trauma care pathways.

The inclusion criteria were as follows: (1) working in a trauma center–certified hospital; (2) having ≥ 6 months experience in caring for patients with traumatic injuries; (3) holding a nursing professional qualification certificate with at least 1 year of work experience; and (4) providing informed consent and voluntarily participating in this study. The exclusion criteria included the following: (1) being absent from work for ≥ 3 months during the study period due to maternity leave, further training, or other reasons and (2) being an intern, trainee, or resident in standardized training.

Data collection was conducted through an anonymous web-based questionnaire platform (https//:www.wjx.cn), which employs IP encryption to ensure participant confidentiality. The researchers coordinated with the head nurses of the relevant departments at the participating hospitals. These head nurses received specific training on the study's inclusion and exclusion criteria. They were then responsible for identifying eligible nurses based on these criteria and distributing the survey links directly to the corresponding participants. Prior to questionnaire access, all participants were presented with an introductory statement explicitly affirming the voluntary nature of participation, their right to refuse involvement without consequence, and guaranteed anonymity. The first survey item functioned as a mandatory consent gatekeeper: respondents selected either “Yes” (to proceed to the full survey) or “No” (to immediately exit the platform). Only those opting for “Yes” advanced to subsequent questions, thus establishing documented informed consent prior to data collection.

The sample size for this study was calculated based on the requirements for multiple linear regression analysis. Considering that 14 items were included as potential predictors, with a recommended sample size of 5–10 times the number of items, a minimum of 140 nurses were required to participate in this study.

### 2.3. Instruments

From an implementation science perspective [33], nurses' TIC-KAP reflects their current engagement level with TIC principles, which may be shaped by interconnected factors across four domains: (1) individual attributes, (2) inner setting (e.g., unit-level resources/climate), (3) outer setting (e.g., hospital policies/reimbursement), and (4) implementation processes. Guided by the Consolidated Framework for Implementation Research (CFIR) [33], we conceptualize nurses' TIC-KAP as an indicator of TIC implementation fidelity at the nurse level, which interacts with four interdependent domains: the inner setting (organizational culture and clinical workload), outer setting (department/ward classification), individual characteristics (demographics including gender, age, education level, and professional title; psychological attributes such as empathy; and prior TIC knowledge), and implementation process (availability of TIC-specific training programs and dedicated psychological nursing positions). Therefore, this study utilized a self-designed general information questionnaire, TIC-KAP questionnaire, the Chinese version of the Interpersonal Reactivity Index (IRI-C), and the Organizational Climate Scale for Nursing (OCSN) to conduct the survey.

The general information questionnaire included items related to the nurse's department, age, gender, ethnicity, marital status, educational level, professional title, years of work experience, number of night shifts per month, weekly working hours, whether they had heard of TIC, whether they had participated in any training related to psychological trauma, and whether their department had dedicated psychological nursing positions.

The TIC-KAP questionnaire was developed by Xia et al. [34] to measure TIC-related competencies of nurses caring for trauma patients through 30 items across three dimensions: knowledge, attitude, and practice. All items are rated using a Likert scale with scores ranging from 1 to 5. During the scale development process, the content validity was 0.971, and the Cronbach's alpha coefficient was 0.939, indicating good reliability and validity. In this study, the overall Cronbach's alpha coefficient of the scale was 0.963.

The IRI-C is derived from the original Interpersonal Reactivity Index (IRI) developed by Davis [35] to measure an individual's empathy level. The IRI-C was translated into Chinese and has been validated and revised specifically for Chinese-speaking groups by Zhan et al. [36, 37]. The IRI-C consists of four factors and 22 items, scored using a Likert scale ranging from 0 to 4. In the general population, the IRI-C has demonstrated good reliability, with a Cronbach's alpha coefficient of 0.751 and a test–retest reliability of 0.770. In this study, the Cronbach's alpha coefficient for the IRI-C was 0.764.

The OCSN was developed by He et al. [38] to measure the organizational climate perceived by nurses. The scale comprises 34 items across four dimensions: fair supportive behaviors, colleague behaviors, interpersonal climate behaviors, and close and proactive climate behaviors. It uses a Likert scale with values ranging from 1 to 5. The Cronbach's alpha coefficient for this scale was reported to be 0.927, with item-factor correlations ranging from 0.577 to 0.819. In this study, the Cronbach's alpha coefficient for the OCSN was 0.977.

### 2.4. Ethical Considerations

Ethical approval for this study was obtained from the Biomedical Research Ethics Committee of West China Hospital, Sichuan University (No. 2024-1953). Prior to the commencement of the survey, the researchers informed the participating nurses about the method of completing the questionnaire and explicitly stated that refusal to participate would have no adverse impact on them. All data collection was conducted anonymously.

### 2.5. Statistical Analysis

Data analysis was performed using SPSS Statistics 29.0 (IBM Corp., Armonk, NY, USA). Categorical data were described using frequencies and percentages (*n* %). For continuous data, the *Kolmogorov*–*Smirnov* (KS) test was initially used to assess normality. Since the data did not meet the criteria for normal distribution, they were described using the median and interquartile range *M (P25, P75)*. Comparisons of continuous data between groups were conducted using the *Kruskal*–*Wallis H* test. Associations between continuous variables were analyzed using *Spearman's* rank correlation analysis. Finally, multiple linear regression analysis was employed to identify the determinants of TIC-KAP. The significance level was set at alpha = 0.05.

## 3. Results

A total of 304 questionnaires were collected from the 11 hospitals. After the researchers reviewed the content of the questionnaires, 37 were excluded due to completion times of less than 3 min and patterned responses. Consequently, 267 questionnaires were included in the data analysis, yielding an effective rate of 87.83%.

To confirm the reliability of the scale results, a common method bias test was conducted. All scale items were entered into a factor analysis, and principal component analysis was used to extract 10 principal components. The variance explained by the first common factor was 31.83%, which is less than 40%. Therefore, we concluded that there was no serious common method bias.

Table 1 presents the basic characteristics of the nurses, as well as their scores on TIC-KAP, IRI-C, and OCSN. The respondents included in the data analysis were primarily from general surgery (31.84%), emergency department (22.10%), and trauma center (11.99%). As all study sites were tertiary hospitals, institutional level was excluded from analytical considerations. The majority were female (243, 91.01%), with a median age of 33 (28, 38) years. The median TIC-KAP score was 118 (107, 133). Specifically, among the dimensions of TIC-KAP, the knowledge dimension had the lowest score, followed by attitude and practice (median item scores: 3 for knowledge vs. 4 for attitude vs. 4 for practice). For detailed descriptions of the scores across each dimension of TIC-KAP, see Appendix 2 Table 1.

Table 2 presents univariate analysis results. Significant differences emerged in TIC knowledge and TIC-KAP total scores across departments, with emergency nurses demonstrating the lowest total scores (45 [38, 50]). Variations in TIC knowledge were also observed among ethnic groups and marital status categories, though no differences in overall TIC-KAP scores reached significance. Additionally, prior knowledge of TIC significantly influenced both TIC knowledge (*p*=0.002) and total KAP scores (*p*=0.009). Psychological trauma nursing training experience and dedicated psychological nursing positions showed significant positive effects across all three TIC-KAP dimensions.

Table 3 shows the correlation between age, IRI-C, OCSN, and TIC-KAP. Age was not significantly correlated with the total TIC-KAP score. However, both IRI-C and OCSN were significantly correlated with the total TIC-KAP score (*p* < 0.001).

A linear regression model was constructed with total TIC-KAP scores as the dependent variable, incorporating all factors demonstrating statistically significant associations in univariate analyses as independent variables (Table 4). The model explained 39.7% of score variance (*adjustedR*^2^ = 0.373, *F* = 16.823, *p* < 0.001). Significant positive predictors of KAP scores included the following: prior knowledge of TIC (*β* = 3.707, *p*=0.040), participation in psychological trauma training (*β* = 4.519, *p*=0.048), availability of dedicated psychological nursing positions (*β* = 6.586, *p*=0.003), higher IRI-C scores (*β* = 0.600, *p* < 0.001), and higher OCSN scores (*β* = 0.443, *p* < 0.001). Conversely, working in emergency department inversely predicted KAP scores (*β* = −6.252, *p*=0.027).

## 4. Discussion

This study surveyed nurses caring for traumatic injured patients to assess their knowledge, attitude, and practice regarding TIC and to identify key influencing factors. Results indicated that nurses' TIC knowledge levels were lower than their attitude and practice levels. Furthermore, TIC-KAP scores were influenced by several factors, including working departments, prior knowledge of TIC, psychological trauma nursing training experience, availability of dedicated psychological nursing positions, individual empathy levels, and unit-level organizational climate.

### 4.1. TIC-KAP Scores Among Nurses Caring Traumatic Injured Patients

The survey revealed that clinical nurses generally endorsed the principles of TIC, but their knowledge levels were relatively low (median scores: 4 for attitude items vs. 3 for knowledge items). Even mental health professionals often demonstrate limited knowledge of TIC principles [24]. This competency gap appears prevalent across diverse healthcare provider populations [29, 39]. For instance, a survey of obstetrics residents in the United States and Canada found that only 20% received annual TIC-related training, 53% had training less than once per year, and 27% had never been exposed to any related training [40]. The deficiency in both theoretical and practical knowledge prevents healthcare providers from effectively screening, identifying, and intervening for trauma survivors [41, 42].

### 4.2. Correlations Between Departments and TIC-KAP Scores

This study found that among various departments that receive trauma patients, emergency nurses exhibited lower levels of TIC-KAP, with a particularly significant gap in attitude and practice. This may be related to the shorter turnaround times in the emergency department, the diverse and critical nature of patient conditions, and the higher prevalence of empathy fatigue among emergency nurses [43, 44]. The high-intensity clinical environment in emergency departments may constrain both the time available for TIC delivery and nurses' access to specialized TIC training opportunities [45], which may help explain the relatively lower TIC-KAP scores observed among nurses working in ED settings. These factors may collectively reduce their willingness and actual implementation of TIC. However, it is important to note that trauma is highly prevalent among emergency patients, with over 60% of patients having a history of trauma [46]. Implementing training on TIC may enhance emergency nurses' trauma-informed awareness. When combined with a set of fundamental TIC measures, this approach could help reduce the complexity of implementing TIC in practice, thereby improving nurses' adherence to TIC principles in their clinical work [47].

### 4.3. Correlations Between Previous TIC Knowledge, Psychological Trauma Nursing Training Experience, and TIC-KAP Scores

From an organizational perspective, personnel training is an essential component for improving TIC. This study found that previous TIC knowledge and psychological trauma nursing training experience positively impact TIC-KAP. This is consistent with the earlier research by Ross et al. [48, 49], in which the importance of training was widely acknowledged by healthcare providers in their interviews. Quantitative studies have also supported TIC training effectively enhances healthcare professionals' positive attitude toward TIC, with nursing students showing particularly notable improvements [50]. This training effect is not merely short-lived; improvements in TIC knowledge and attitude at both the individual and organizational levels can still be observed 6 months posttraining [51], further highlighting that effective training is a primary prerequisite for the implementation of TIC.

### 4.4. Correlations Between Institutional Psychological Nursing Positions and TIC-KAP Scores

At the same time, having dedicated psychological nursing positions in nonpsychiatric specialties, exemplified by programs like the “Sunshine Angels” initiative [52], can significantly enhance clinical nurses' comprehension and application of TIC through collaborative practice, interdisciplinary communication, and peer education. The institutional creation of such specialized roles itself reflects organizational commitment to patient psychological care. Hall et al. [53] demonstrated that consistent application of TIC principles by healthcare staff not only promotes patient mental health but also benefits the psychological well-being of themselves and other interdisciplinary team members. We believe that establishing psychological nurse positions within hospitals could enhance healthcare staff's understanding of TIC and promoting its broader implementation.

### 4.5. Correlations Between Empathy Levels and TIC-KAP Scores

Our findings indicate that nurses' empathy levels (IRI-C scores) are directly correlated with their TIC-KAP. Demonstrating empathy toward patients is the first step in identifying their exposure to psychological trauma [54]. Higher levels of empathy among nurses can reduce patients' distress and anxiety and increase the likelihood of recognizing the needs of both patients and caregivers [55]. In studies on TIC training, trainees have been shown to exhibit increased empathy toward patients [56]. Cultivating nurses' empathy may be a promising approach to improve TIC-KAP. Some training methods that place greater emphasis on direct experience and emotional resonance, such as standardized patient simulation [57] and digital story [58], have been recognized as effective approaches for enhancing nurses' empathy.

### 4.6. Correlations Between Organizational Climate and TIC-KAP Scores Among Nurses

Moreover, the organizational climate in the workplace (OCSN scores) was significantly correlated with TIC-KAP scores. This association may be attributed to the fact that a positive organizational climate can enhance nurses' psychological resilience, thereby facilitating the implementation of TIC in clinical practice [59]. This possibility is corroborated by the research of Damian et al. [60], who argued that TIC training should focus on organizational factors, such as providing a safe atmosphere, high morale, and team collaboration, to better support the implementation of TIC. Similarly, Mahon [61] noted that a positive organizational climate can empower employees and foster better collaboration in shared decision making, both of which are conducive to the implementation of the trustworthiness principle in TIC. Nursing managers can foster a positive organizational climate through targeted strategies such as setting team goals, fostering teamwork, managing organizational change, providing counseling services, and establishing multilevel feedback mechanisms [62, 63]. A climate built on these foundations directly contributes to enhancing nurses' personal resilience, thereby strengthening their confidence and willingness to implement TIC.

### 4.7. Study Limitations

This study has several limitations that should be acknowledged. First, the investigation was confined to multiple trauma centers within Sichuan Province, excluding nurses from other geographical regions across China. Second, considering that the surveyed nurses were employed in trauma center–certified hospitals, their levels of TIC-KAP may be comparatively higher than those working in non-trauma center hospitals, which still constitute a substantial proportion of healthcare facilities in China. Future research should examine the potential influence of trauma center designation on TIC-KAP levels. Third, the 91.01% (186/243) female representation in our sample aligns with China's nursing workforce demographics (96.5% female per 2023 national data) [64], mitigating concerns about gender selection bias despite potential influence on results. Finally, while this study focused on specific factors of interest, the potential impact of additional unexplored variables remains unknown. Subsequent investigations should incorporate expanded sample sizes and include nurses from diverse regions and various hospital types to enhance the generalizability of findings.

## 5. Conclusion

The findings of this study indicate that the level of TIC provided by nurses to traumatic injured patients requires further improvement, with particular attention needed in emergency department settings. The investigation revealed that several factors significantly influence nurses' knowledge, attitude, and practice regarding TIC, including prior awareness of TIC concepts, participation in psychological training programs, establishment of dedicated psychological nursing positions, nurses' empathy levels, and organizational climate. To enhance TIC implementation, future initiatives should focus on expanding training opportunities, increasing organizational attention and advocacy for TIC at the institutional level, and improving workplace environments to foster better adoption of TIC practices.

## Figures and Tables

**Table 1:** Basic characteristics of respondents (*N* = 267).

Characteristic		*M* (P25, P75)/*n* (%)
Ward	Trauma center	32 (11.99)
	Emergency department	59 (22.10)
	Orthopedics	26 (9.74)
	Neurosurgery	16 (5.99)
	General surgery	85 (31.84)
	Other	49 (18.35)

Gender	Male	24 (8.99)
	Female	243 (91.01)

Age		33 (28.38)

Ethnicity	Han	212 (79.40)
	Other	55 (20.60)

Marital status	Unmarried	74 (27.72)
	Married	183 (68.54)
	Divorced	10 (3.75)

Education level	Associate degree	45 (16.85)
	Bachelor's degree	218 (81.65)
	Postgraduate	4 (1.50)

Professional title	Nurse	30 (11.24)
	Senior nurse	120 (44.94)
	Supervisor nurse	93 (34.83)
	Associate chief nurse or above	24 (8.99)

Years of working	1–5 years	65 (24.34)
	6–10 years	71 (26.59)
	11–20 years	101 (37.83)
	> 20 years	30 (11.24)

Night shifts per month	None	55 (20.60)
	1–3	64 (23.97)
	4–7	88 (32.96)
	> 7	60 (22.47)

Work hours per week	≤ 40 h	92 (34.46)
	40–50 h	153 (57.30)
	> 50 h	22 (8.24)

Have you ever heard of trauma-informed care before this survey?	Yes	140 (52.43)
	No	127 (47.57)

Have you participated in training related to topics such as psychological trauma, posttraumatic stress disorder, adverse childhood experiences, or trauma-informed care?	Yes	78 (29.31)
	No	189 (70.79)

Does your hospital have dedicated positions for psychological care nurses in non-mental health–related departments?	Yes	107 (40.07)
	No	160 (59.93)

TIC-KAP score		118 (107.133)

IRI-C score		52 (46.57)

OCSN score		101 (95.116)

*Note:* Professional titles in Chinese nursing reflect hierarchical competency levels. Newly qualified nurses are designated as “Nurse.” With several years of clinical experience and successful completion of certification examinations, nurses may advance to “Senior Nurse” or “Supervisor Nurse” positions. Attaining the “Associate Chief Nurse” title or higher requires demonstrated integration of clinical experience, research capabilities, teaching competencies, and examination performance.

Abbreviations: IRI-C, Chinese version of the Interpersonal Reactivity Index; OCSN, Organizational Climate Scale for Nursing; TIC-KAP, trauma-informed care knowledge, attitude, and practice.

**Table 2:** Differences of TIC-KAP scores among nurses with different characteristics.

	Knowledge	*p* value	Attitude	*p* value	Practice	*p* value	Total score	*p* value
Ward		**<** **0.001**		0.583		0.121		**0.015**
Trauma center	32 (31, 39.3)		46 (40, 50)		49 (45, 59)		128.5 (118, 144.3)	
Emergency department	28 (24, 32)		42 (40, 48)		45 (38, 50)		114 (104.5, 128.0)	
Orthopedics	28 (24, 32)		45.5 (40, 48.8)		48 (41.8, 54.3)		118.5 (107, 134)	
Neurosurgery	28.5 (24.8, 32)		48 (40, 50)		52.5 (47.8, 59.3)		126 (109.3, 139)	
General surgery	25 (22, 32)		43 (40, 50)		48 (42, 58)		116 (106, 129)	
Other	27 (24, 32)		44 (40.50)		48 (45, 56)		120 (112, 131)	
Gender		0.569		0.948		0.914		0.663
Male	30.5 (24, 32)		43.5 (40, 49)		48.5 (41, 56)		121.5 (110, 131.5)	
Female	28 (24, 32)		43 (40, 50)		48 (42, 56)		118 (107, 133)	
Ethnicity		**0.014**		0.776		0.582		0.113
Han	29 (24, 32)		43 (40, 49)		48 (42, 56.3)		119 (107, 134)	
Other	25 (22.5, 31)		44 (40, 50)		48 (38.5, 56)		113 (105, 130)	
Marital status		**0.042**		0.693		0.727		0.585
Unmarried	31 (26, 32)		43 (40, 49.8)		48 (42.3, 56)		118.5 (107.3, 136.8)	
Married	26 (24, 32)		44 (40, 50)		48 (42, 56.5)		118 (107, 132.5)	
Divorced	24 (24, 39.5)		41.5 (40, 45.5)		45.5 (36, 54.3)		109.5 (101.8, 130)	
Education level		0.059		0.933		0.703		0.404
Associate degree	28 (24.32)		44 (40, 50)		48 (43, 60)		118 (108, 133)	
Bachelor's degree	28 (24, 32)		43 (40, 49)		48 (41, 56)		118 (107, 132.8)	
Postgraduate	36 (32, 40)		44.5 (38.5, 50)		47 (47, 50)		127.5 (117.5, 140.3)	
Professional title		0.146		0.641		0.460		0.297
Nurse	31 (25.3, 32)		40 (40, 50)		48 (45.3, 60)		118 (112.3, 140.5)	
Senior nurse	29 (24, 32)		44 (40, 50)		48 (42.8, 56)		119.5 (108, 133)	
Supervisor nurse	26 (23, 32)		42 (40, 48)		48 (40, 52)		113 (104, 132)	
Associate chief nurse or above	29 (24, 32.5)		45 (40, 48)		48 (40, 57)		119 (107, 132.3)	
Years of working		0.088		0.859		0.827		0.415
1–5 years	31 (25, 32)		44 (40, 50)		48 (44, 58)		120 (110, 136)	
6–10 years	27 (24, 32)		43 (40, 49)		48 (40, 56)		118 (107.5, 130)	
11–20 years	26 (23, 32)		43 (40, 49)		48 (41, 55)		114 (104, 134)	
> 20 years	29 (24.3, 32.8)		45.5 (40, 48)		48 (42.8, 55.8)		122 (110.5, 133)	
Night shifts per month		0.391		0.278		0.251		0.544
None	29 (24, 34.5)		45 (40, 50)		48 (42, 60)		121 (106, 138.5)	
1–3	27.5 (23.8, 32)		43 (40, 50)		48 (44, 52)		119 (107, 131)	
4–7	27.5 (24, 32)		44.5 (40, 50)		48 (42.8, 60)		118 (110, 139)	
> 7	29 (24, 32)		40 (40, 48.3)		48 (37.8, 53.5)		116.5 (106, 128.5)	
Work hours per week		0.095		0.142		0.387		0.094
≤ 40 h	29.5 (24, 33)		45 (40, 50)		48 (42.8, 58.3)		120 (110.3, 139)	
40–50 h	28 (24, 32)		43 (40, 49)		48 (41, 56)		116 (106, 131)	
> 50 h	25 (23.3, 31.8)		40 (40, 46)		48 (41.3, 52)		115.5 (107.5, 126)	
Have you ever heard of trauma-informed care before this survey?		**0.002**		0.389		0.073		**0.009**
Yes	30 (24, 32.3)		43.5 (40, 50)		48 (44, 58)		120 (109, 137)	
No	25 (22, 32)		43 (40, 49)		48 (39.5, 55)		116 (105, 129)	
Have you participated in training related to topics such as psychological trauma, posttraumatic stress disorder, adverse childhood experiences, or trauma-informed care?		**< 0.001**		**0.014**		**0.004**		**< 0.001**
Yes	32 (28.3, 38)		47 (40, 50)		49.5 (45, 60)		130 (112, 143.5)	
No	25 (23, 32)		42 (40, 49)		48 (40, 54)		114 (104, 129)	
Does your hospital have dedicated positions for psychological care nurses in non-mental health–related departments?		**< 0.001**		**0.004**		**0.002**		**< 0.001**
Yes	32 (28, 35.5)		46 (40, 50)		48 (45, 60)		126 (112.5, 142)	
No	24 (22, 31)		41 (40, 48)		48 (39, 53)		113 (103.8, 128.3)	

*Note:* Bold values represent the results with a *p* value of less than 0.05.

**Table 3:** Correlations of age, IRI-C, OCSN, and TIC-KAP scores.

	Age	IRI-C	OCSN
*TIC-KAP*			
*r*	−0.061	0.391	0.474
*p*	0.324	**< 0.001**	**< 0.001**

*Note:* Bold values represent the results with a *p* value of less than 0.05.

Abbreviations: IRI-C, Chinese version of the Interpersonal Reactivity Index; OCSN, Organizational Climate Scale for Nursing; TIC-KAP, trauma-informed care knowledge, attitude, and practice.

**Table 4:** Multivariate regression analysis of TIC-KAP scores.

Predictors	*β*	SE	*t*	*p*
Intercept	38.656	7.979	4.845	**< 0.001**
Ward (vs. others)				
Emergency department	−6.252	2.803	−2.231	**0.027**
General surgery	−1.730	2.559	−0.676	0.500
Neurosurgery	3.345	4.108	0.814	0.416
Orthopedics	0.625	3.461	0.181	0.857
Trauma center	−0.750	3.557	−0.211	0.833
Have ever heard of trauma-informed care	3.707	1.796	2.064	**0.040**
Have participated in psychological training	4.519	2.273	1.988	**0.048**
Have dedicated psychological care nurse positions	6.586	2.218	2.969	**0.003**
IRI-C	0.600	0.117	5.113	**<** **0.001**
OCSN	0.443	0.064	6.947	**<** **0.001**

*Note:* Bold values represent the results with a *p* value of less than 0.05.

Abbreviations: IRI-C, Chinese version of the Interpersonal Reactivity Index; OCSN, Organizational Climate Scale for Nursing; TIC-KAP, trauma-informed care knowledge, attitude, and practice.

## Data Availability

The data that support the findings of this study are available from the corresponding authors upon reasonable request.

## Supporting Information

Supporting Information 1 Appendix 1. STROBE checklist. This file presents the Strengthening the Reporting of Observational Studies in Epidemiology (STROBE) checklist for this study.

Supporting Information 2 Appendix 2. Table 1. This file presents survey results about the performance metrics of respondents on specific domains within the TIC-KAP questionnaire.
