# Supplementary material for: The Knowledge, Attitude, and Practice of Nurses Regarding Trauma-Informed Care for Traumatic Injured Patients: A Multicenter Cross-Sectional Study
Source: J Nurs Manag. 2025 Nov 25;2025:2449177. doi: 10.1155/jonm/2449177 (PMC12672082; doi:10.1155/jonm/2449177)
Supplement: Supporting Information 2 — Appendix 2. Table 1. This file presents survey results about the performance metrics of respondents on specific domains within the TIC-KAP questionnaire. [file 2449177.f2.docx]

**Table 1. Performance metrics of respondents on specific domains within the TIC-KAP questionnaire**

| **item of TIC-KAP questionaire** | **M[P25,P75]** |
| --- | --- |
| **Knowledge(8 items)** | 28[24,32] |
| K1. Traumatic events can significantly impact emotional regulation, potentially resulting in symptoms such as uncontrollable crying episodes, anger outbursts, intense fear responses, and emotional numbness. | 3[3,4] |
| K2. Trauma exposure may lead to behavioral alterations, including temper tantrums, impulsive actions, compulsive behaviors, and social withdrawal tendencies. | 4[3,4] |
| K3. Cognitive functioning can be substantially affected by traumatic experiences, manifesting as memory impairment, concentration difficulties, confusion, negative self-perception, and impaired decision-making capacity. | 4[3,4] |
| K4. Traumatic experiences can fundamentally reshape an individual's value system, particularly regarding the prioritization and perception of significant life events. | 3[3,4] |
| K5. Severe traumatic incidents may induce acute stress responses, characterized by either psychomotor arousal (e.g., intense fearful experiences) or psychomotor inhibition (e.g., stupor-like states). | 4[3,4] |
| K6. Acute stress response symptoms typically demonstrate transient duration, generally resolving within several days to one week, representing normative individual reactions to stressful events with favorable prognostic outcomes. | 3[3,4] |
| K7. Significant traumatic events may precipitate post-traumatic stress disorder (PTSD), characterized by persistent symptoms including intrusive recollections, avoidance behaviors, chronic anxiety, and hypervigilance, potentially accompanied by maladaptive coping mechanisms such as substance abuse, aggressive behaviors, self-harm tendencies, or suicidal ideation. | 3[3,4] |
| K8. Post-traumatic outcomes demonstrate considerable variability, with some individuals achieving rapid psychosocial recovery or even post-traumatic growth, while others may experience enduring adverse effects on psychosocial functioning. | 3[3,4] |
| Median item scores | 3 |
| **Attitude(10 items)** | 43[40,50] |
| A1. It is essential to provide care for trauma patients by alleviating their psychological distress and fostering a sense of psychological safety. | 5[4,5] |
| A2. Healthcare providers must respect trauma patients' diverse backgrounds, including cultural needs, ethnicity, gender, age, religious beliefs, and personal values. | 5[4,5] |
| A3. Comprehensive trauma-related information should be obtained to facilitate appropriate medical interventions for trauma patients. | 5[4,5] |
| A4. Addressing trauma patients' psychological concerns requires a multidisciplinary approach involving nurses, physicians, psychiatrists, counselors, social workers, and family members. | 5[4,5] |
| A5. Healthcare institutions should implement comprehensive training programs in trauma-informed care for nursing staff. | 4[4,5] |
| A6. There is a professional imperative to acquire proficiency in psychological trauma assessment and screening instruments for trauma patients. | 4[4,5] |
| A7. Continuous access to current evidence-based research and developments in trauma-informed care is crucial for clinical practice. | 4[4,5] |
| A8. Trauma patients possess inherent resilience and coping mechanisms that should be recognized and supported. | 4[4,5] |
| A9. Mastery of evidence-based psychological interventions for trauma patients is essential for optimal patient care. | 4[4,5] |
| A10. Healthcare providers should demonstrate understanding and empathy toward the emotional responses exhibited by trauma patients | 4[4,5] |
| Median item scores | 4 |
| **Practice(12 items)** | 48[42,56] |
| P1. I learn from patients or families about their trauma experiences in a respectful and professional manner without re-traumatizing them. | 4[4,5] |
| P2. I assess whether the traumatically injured patient has the past trauma history, mental disease, substance abuse, or other potential risk factors for post-traumatic stress disorder (PTSD). | 4[3,5] |
| P3. After the traumatic injury, I assess the patient’s psychological state by observation, talking or psychological scales to access the psychological impact. | 4[3,5] |
| P4. As taking care of traumatically injured patients with psychological trauma, I avoid irritate them with inappropriate words and behaviors. | 4[4,5] |
| P5. In a propriate time, I instruct the traumatically injured patients or their family members how to seek professional services such as psychiatric consultation, psychotherapy, psychological counselling, etc. | 4[4,5] |
| P6. I help traumatically injured patients to get peer support (face-to-face, internet, symposium). | 4[4,5] |
| P7. I teach traumatically injured patients relaxation techniques to relieve their negative emotions (eg, deep breathing, muscle relaxation, meditation). | 4[3,5] |
| P8. I give instructions for the traumatically injured patients or their family about the symptoms and recovery process of traumatic stress reactions. | 4[3,5] |
| P9. I assess the cultural needs of traumatically injured patients and provide culturally sensitive care. | 4[3,5] |
| P10. I avoid denying or blaming the traumatically injured patient’s emotions. | 4[3,5] |
| P11. I explore and acknowledge the positive resources of the traumatically injured patients, such as important person in life, will to survive, strong character traits, resilient character, hobbies, etc. | 4[3,5] |
| P12. I collaborate with professionals, such as counselors, psychiatrists, social workers, and help provide necessary referrals for traumatically injured patients as needed. | 4[3,5] |
| Median item scores | 4 |
